# *Plasmodium ovale curtisi* and *Plasmodium ovale wallikeri* circulate simultaneously in African communities^☆^

**DOI:** 10.1016/j.ijpara.2011.01.004

**Published:** 2011-05

**Authors:** Mary Chiaka Oguike, Martha Betson, Martina Burke, Debbie Nolder, J. Russell Stothard, Immo Kleinschmidt, Carla Proietti, Teun Bousema, Mathieu Ndounga, Kazuyuki Tanabe, Edward Ntege, Richard Culleton, Colin J. Sutherland

**Affiliations:** a Department of Immunology and Infection, Faculty of Infectious & Tropical Diseases, LSHTM, UK; b Wolfson Wellcome Biomedical Labs, Department of Zoology, Natural History Museum, London, UK; c HPA Malaria Reference Laboratory, Faculty of Infectious & Tropical Diseases, LSHTM, UK; d Department of Infectious Disease Epidemiology, Faculty of Environmental & Population Health, LSHTM, UK; e Centre d’Etudes des Resources Vegetales, Brazzaville, Republic of Congo; f Laboratory of Malariology, International Research Centre of Infectious Diseases, Research Institute of Microbial Diseases, Osaka University, Osaka, Japan; g Medical Biotech Laboratories, Kampala, Uganda; h Department of Protozoology, Institute of Tropical Medicine (NEKKEN) and The Global Center of Excellence Program, Nagasaki University, Nagasaki, Japan

**Keywords:** **Plasmodium ovale curtisi**, **Plasmodium ovale wallikeri**, Sympatry

## Abstract

It has been proposed that ovale malaria in humans is caused by two closely related but distinct species of malaria parasite, *Plasmodium ovale curtisi* and *Plasmodium ovale wallikeri*. It was recently shown that these two parasite types are sympatric at the country level. However, it remains possible that localised geographic, temporal or ecological barriers exist within endemic countries which prevent recombination between the genomes of the two species. Here, using conventional and real-time quantitative PCR (qPCR) methods specifically designed to discriminate *P. o. curtisi* and *P. o. wallikeri*, it is shown that both species are present among clinic attendees in Congo-Brazzaville, and occur simultaneously both in lake-side and inland districts in Uganda and on Bioko Island, Equatorial Guinea. Thus *P. o. curtisi* and *P. o. wallikeri* in these localities are exactly sympatric in both time and space. These findings are consistent with the existence of a biological barrier, rather than geographical or ecological factors, preventing recombination between *P. o. curtisi* and *P. o. wallikeri*. In cross-sectional surveys carried out in Uganda and Bioko, our results show that infections with *P. ovale* spp. are more common than previously thought, occurring at a frequency of 1–6% in population samples, with both proposed species contributing to ovale malaria in six sites. Malaria elimination programmes in Africa need to include strategies for control of *P. o. curtisi* and *P. o. wallikeri*.

## Graphical abstract

**Graphical abstract:**

**Figure:**
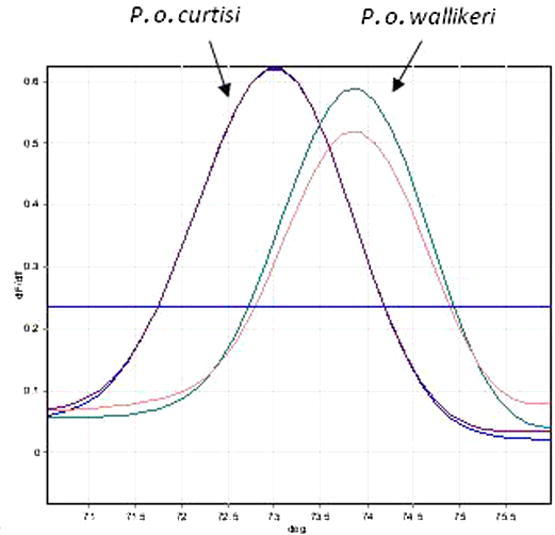

**Research highlights:**

► We propose that two related species of malaria parasite cause ovale malaria. ► Discriminatory PCR tests identified both species in African surveys. ► *Plasmodium ovale curtisi* and *Plasmodium ovale wallikeri* were found together in time and space. ► Lack of recombination between them is not due to geographic or temporal separation. ► Therefore, the species barrier may be maintained by a biological mechanism(s).

## 1 Introduction

The human malaria agent *Plasmodium ovale* was described by Stevens in 1922. Since that time, relatively little attention has been paid to ovale malaria, which is considered to be uncommon, mild in clinical presentation and easily treated with the conventional antimalarial drug chloroquine (Mueller et al., 2007). Difficulties in performing definitive species-level diagnosis in endemic settings are likely to have resulted in a systematic under-estimation of the number of cases of ovale malaria across its tropical range; analysis of UK reference laboratory data over a 20 year period strongly suggests that a substantial burden of ovale malaria occurs in sub-Saharan Africa (Sutherland et al., 2010). *Plasmodium ovale* has also proved difficult to identify using species-specific molecular tests, as a sizeable subset of isolates do not produce the expected DNA amplification products with established ovale-specific primers targeting the ssrRNA gene, and may be missed by standard rapid diagnostic tests (Calderaro et al., 2007; Talman et al., 2007). Sequencing of rRNA genes from two well-characterised isolates of *P. ovale* first prompted the suggestion that this species was dimorphic, and raised the possibility that two sub-species might exist (Li et al., 1995). Analysis of loci other than rRNA genes supported the idea that this dimorphism was multigenic, leading to the concept of “variant” ovale parasites (Tachibana et al., 2002; Win et al., 2004; Calderaro et al., 2007). In a study of *P. ovale* conducted by researchers from Mahidol University, Bangkok, and the UK Malaria Reference Laboratory (UKMRL), London, polymorphisms in six loci were examined in 55 isolates. Two distinct major haplotypes of each locus were identified and these did not recombine in any of the parasites examined. Thus *P. ovale* dimorphism was proposed to reflect the existence of two fully distinct ovale malaria species, which were unexpectedly shown to be broadly sympatric, at the country level, in both Africa and Asia (Sutherland et al., 2010). These two proposed species have been named *Plasmodium ovale curtisi* and *Plasmodium ovale wallikeri*.

The recognition that two separate parasite species may be the agents of ovale malaria raises many questions, but perhaps the most intriguing puzzle is the unknown nature of the mechanism that keeps these parasites genetically separate, when they are found in the same countries in Asia and in Africa. The elucidation of any biological/genetic mechanism or mechanisms separating *P. o. curtisi* and *P. o. wallikeri* requires detailed investigation of the biology of these two pathogens, which is hampered by the lack of a system for in vitro cultivation and thus requires in vivo and ex vivo approaches. Physical separation is a plausible non-biological barrier to recombination and could be caused by geographic discontinuities in within-country distribution or temporal causes such as adaptation to transmission at different times of the year. Such physical barriers could conceivably prevent both species from being ingested by a feeding *Anopheles* mosquito in a single bloodmeal, the prerequisite for meiotic recombination. We endeavored to determine whether both *P. o. curtisi* and *P. o. wallikeri* could be identified in the same community at the same time, using samples from febrile malaria patients presenting to clinics in Congo-Brazzaville, or from cross-sectional parasitological sampling over one or a few days in single communities in Uganda and in Bioko Island, Equatorial Guinea. This parasite material was collected in the course of studies of other *Plasmodium* spp. but in each site a measurable contribution of *P. o. curtisi* and *P. o. wallikeri* to the overall prevalence of parasitaemia was demonstrated.

## 2 Materials and methods

### 2.1 Parasite sample collection and DNA extraction

#### 2.1.1 Congo-Brazzaville

Blood samples were taken from febrile patients attending health centres in three separate locations within the Republic of Congo between 2005 and 2007 as previously described (Culleton et al., 2008, 2009). Sampled health centres include Madibou and Tenrikyo within Brazzaville, the capital of the Republic of Congo, Mbota in Pointe-Noire (a city on the west coast of the country) and Gamboma, a town in the east. Samples collected in 2005 were collected on Whatman® FTA® filter paper and processed as previously described (Culleton et al., 2008). All remaining samples were collected on Whatman® 31ETCHR filter paper and DNA extraction was performed using the EZ1 BioRobot™ (QIAGEN, Hilden, Germany) according to the manufacturer’s instructions. Results of initial PCR species diagnoses, performed on all samples except those from Pointe-Noire in 2007, which were diagnosed by microscopy alone, have been presented previously (Culleton et al., 2008). *Plasmodium ovale-*positive purified DNA samples were shipped to London, UK for this study.

#### 2.1.2 Buliisa and Mayuge districts, Uganda

Single finger-prick blood samples were collected onto Whatman® 3M filter paper from 1850 mothers and young children enrolled in a longitudinal study of schistosomiasis and malaria infection in six lakeside communities (Bugoigo, Walukuba, Piida, Bugoto, Bukoba and Lwanika) in Buliisa and Mayuge Districts, Uganda (Betson et al., 2010). Samples from each village were collected over a period of 3–5 days. Samples were transported to the London School of Hygiene and Tropical Medicine, (LSHTM), UK for DNA extraction by a modification of the Chelex method, as previously described (Dlamini et al., 2010), and were screened using a species-specific probe-based real-time PCR assay in order to detect *P. ovale* parasite DNA for testing in the *curtisi – wallikeri* discrimination assay (Shokoples et al., 2009).

#### 2.1.3 Apac district, northern Uganda

A health facility-based survey was conducted in the parish of Abedi in Apac district, northern Uganda. This area is hyperendemic for *Plasmodium falciparum* malaria with an estimated entomological inoculation rate >1000 infectious bites per person per year (Okello et al., 2006). All people attending a local health facility, regardless of reasons for presentation, were asked to donate a small blood sample for microscopic slide preparation and blood spot collection on filter paper (Whatman® 3MM). DNA was extracted from 241 samples, with a microscopical prevalence of *P. falciparum* of 37.6% (89/237) and no other parasite species were identified by microscopy. DNA was extracted by the Chelex Method (Dlamini et al., 2010) for microscopically positive and negative samples.

#### 2.1.4 Bioko Island, Equatorial Guinea

Intensive malaria control interventions have been carried out on Bioko Island, Equatorial Guinea, by The Bioko Island Malaria Control Project (BIMCP) in collaboration with the Ministry of Health and Social Welfare of Equatorial Guinea. As part of the evaluation of the BIMCP, annual malaria indicator surveys have been carried out since 2004 (Kleinschmidt et al., 2009). During the 2009 annual survey, which was carried out across the whole island between July and September of that year, blood samples were collected from 209 individuals in the town of Luba and from 110 individuals in Punta Europa Sacriba. Samples were spotted onto glass-fibre paper (Filtermat A, Wallac, Finland) and shipped to LSHTM, UK for DNA extraction by the Chelex method (Dlamini et al., 2010). Initial screening for the presence of parasites belonging to the genus *Plasmodium* was carried out using the loop-activated amplification (LAMP) assay of Polley et al. (2010). The species present in positive samples were discriminated by the nested PCR of Snounou and colleagues, using a modified primer set to detect all *P. ovale* parasites (Calderaro et al., 2007).

### 2.2 PCR amplification and DNA sequencing

**Fig. 1:**
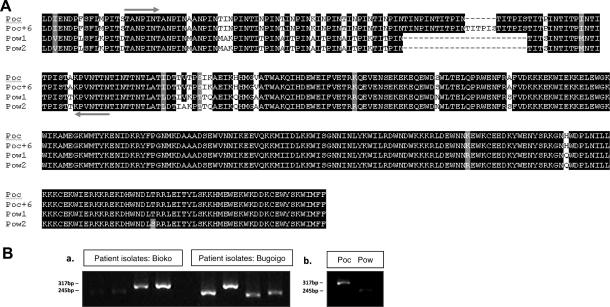
Discrimination between *Plasmodium ovale curtisi* (Poc) and *Plasmodium ovale wallikeri* (Pow) using nested PCR amplification of the repeat region of *poctra* and *powtra* genes. (A) Alignment of the translated amino acid sequence encoded by a large portion of the *P. ovale* sp. tryptophan-rich antigen gene (*potra*), showing the four different forms sequenced (Poc, Poc + 6, Pow1, Pow2). Black shading indicates amino acid identity in at least three sequences, grey shading indicates the occurrence of different amino acids with similar biochemical properties such as charge, size or hydrophobicity, and absence of shading indicates the presence of different amino acids with distinct biochemical properties. Amplification primers (PoTRA5 pair) are situated at the arrows, and are designed to maximise visible size differences in PCR products by gel electrophoresis. Some Nigerian isolates of *P. o. curtisi* have an additional six amino acids in the repeat region (Poc + 6) (Sutherland et al., 2010). (B) Agarose gel showing size distinction between Poc and Pow isolates after a single PCR using PoTRA5 primers. Fragment sizes: *poctra *= 317 bp; *poctra *+ 6 = 335 bp; *powtra *= 245 bp. (a) shows four isolates from Bioko Island, Equatorial Guinea (one Poc from Punta Europa, one Poc from Luba, two Pow from Luba) and four from Bogoigo, Uganda. Previously-characterised isolates of each species from the UK Malaria Reference Laboratory are shown in (b).

Genomic DNA sequences were obtained as previously described (Dlamini et al., 2010) except that removal of incorporated primers and nucleotides from PCR products was performed using the ExoSAP-IT protocol (GE Healthcare). Sequences were aligned in Clustal V (Fig. 1). Conventional PCR and sequencing reactions were performed on a GRI Tetrad thermocycler. For amplification of *potra* (encoding the *P. ovale* sp. tryptophan-rich antigen) loci from field samples, the primers presented in Table 1 and Fig. 1 were used in a nested procedure, with PoTRA3 amplification followed by PoTRA5 amplification.

Real-time amplification assays were performed using the Quantitect Sybr Green PCR kit (Qiagen) on Corbett Rotorgene RG3000 and RG6000 thermo-cyclers, and output was measured by detecting SYBR green incorporation on the FAM fluorescence detection channel. PCR primers and cycling conditions were as previously described (Sutherland et al., 2010) or are given in Table 1.

For a subset of samples, additional amplification and sequencing of the *po3gp* locus, encoding glyceraldehyde-3-phosphatase, was carried out exactly as described (Sutherland et al., 2010) to confirm species designations using a third gene (data not shown).

### 2.3 GenBank accession numbers

The *potra* gene sequences determined specifically for this study were deposited in GenBank with accession numbers HM594180–HM594183 inclusive.

### 2.4 Ethics approval

LSHTM, UK (application No. 5538·09) and the Ugandan National Council of Science and Technology granted ethics approval for the study in Buliisa and Mayuge Districts, Uganda. Informed consent was obtained in writing or by fingerprint (in cases of illiteracy) from all participating mothers on behalf of themselves and their children.

Approval for the Bioko Island Malaria Indicator Survey was given by the ethics committees of the Equatorial Guinea Ministry of Health and Social Welfare, Malabo, and of LSHTM, UK (approval number 5556). Written informed consent was given by each donor, or in the case of young children, by their parent or caregiver.

Ethics approval for the facility-based survey in Apac, Uganda, was obtained from the medical biotechnology laboratories (MBL), the national ethical committee of Uganda and LSHTM, UK (project # 5539.09).

Approval for the collection of blood samples in the Republic of Congo was obtained from the ethics committee at the Research Institute of Microbial Diseases, Osaka University (Osaka, Japan), and sampling was authorised by the administrative authority of the Ministry for Research and the Ministry for Health in the Republic of the Congo. Written informed consent was obtained from individual patients and antimalarial treatment was provided for all individuals diagnosed with clinical malaria.

## 3 Results

### 3.1 Species discrimination by potra nested PCR

To identify a suitable genomic DNA sequence for discrimination between the two species of *P. ovale* on the basis of differential sizes of PCR amplification products, the recently described gene *potra* was amplified from a number of previously characterised *P. o. curtisi* and *P. o. wallikeri* isolates (Sutherland et al., 2010) using the primers and conditions shown in Table 1. Sequences were aligned in Clustal V, and were of four types (Fig. 1A). Two alleles of the *P. o. curtisi* (*poctra*) gene were identified (Poc, Poc + 6), differing in six amino acids in the repeat region, as previously described. Two *P. o. walliker* (*powtra*) alleles were observed (Pow1, Pow2), differing in two non-synonymous positions. A small candidate amplicon encompassing the variable repeat region was delineated and amplified as described in Table 1 (Fig. 1A). Amplified products from a number of clinical and field isolates of *P. ovale* spp*.* were compared by agarose gel electrophoresis, and the two species were clearly distinguishable from each other (Fig. 1B).

### 3.2 Discrimination of poctra and powtra amplicons in ovale malaria cases from Congo

To test the gel-based species discrimination assay, *potra* amplification assays were performed on a group of parasite isolates from symptomatic malaria patients presenting to clinics in coastal (Pointe-Noire), inland (Gamboma) and urban (Brazzaville) locations in the Republic of Congo. In total, 16 isolates had previously been identified as *P. ovale* sp. on the basis of microscopy, and seven additional samples were PCR-positive for *P. ovale* (Culleton et al., 2009). We tested six of these isolates in the nested *potra* assay; there were two *P. o. curtisi* from Brazzaville, the remaining four isolates being identified as *P. o. wallikeri* (Table 2). For each of these six isolates, the result obtained agreed with previous results from amplification of 18S ribosomal sequences conducted at the University of Nagasaki using the method of Snounou (Culleton et al., 2008); microscopically identified ovale parasites failing to amplify using the “classic” primer set were confirmed to be *P. o. wallikeri* by the *potra* discrimination assay.

These results demonstrate that this assay is capable of correctly identifying *P. o. curtisi* and *P. o. wallikeri* in field samples. Both species are present in the Republic of Congo, but the only example of *P. o. curtisi* was found in Brazzaville, more than 200 km from the two sites in which *P. o. wallikeri* was identified. In addition, these isolates were collected over a period of a few years. Therefore these findings do not strictly rule out the possibility of geographic or temporal barriers existing between the two *P. ovale* spp.

### 3.3 Species discrimination by porbp2 quantitative PCR (qPCR) melt profile

**Fig. 2:**
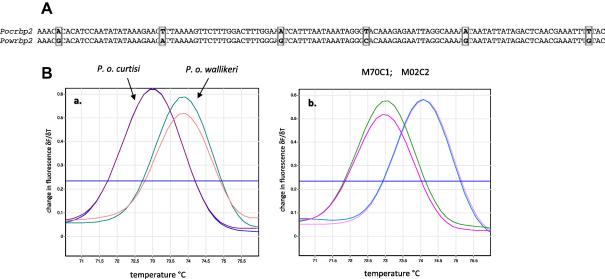
Discrimination between *Plasmodium ovale curtisi* and *Plasmodium ovale wallikeri* using melt profile differences. (A) Alignment of a 120 bp region of the *porbp2* (reticulocyte-binding protein 2) gene between amplification primers Porbp2TMfwd and Porbp2TMrev of *P. o. curtisi* (*pocrbp2*) and *P. o. wallikeri* (*powrbp2*), showing mismatches (boxed) predicted to affect product *T*_m_. (B) Output of melt analysis on a Rotorgene thermo-cycler, expressed as the change in fluorescence over time (δF/δT), displaying clear discrimination between the two species: (a) the amplification product of *P. o. curtisi* exhibits a melt peak at 73 °C, whereas that of *P. o. wallikeri* exhibits a melt peak at 74 °C. (b) Melt profiles of isolates (M70C1, M02C2) from two children sampled in Uganda, each run in duplicate. All controls and samples were run in duplicate.

The genes encoding reticulocyte-binding protein 2 (*porbp2*) in *P. o. curtisi* (*pocrbp2*) and *P. o. wallikeri* (*powrbp2*) (Sutherland et al., 2010) were aligned and compared. A short DNA sequence region with six single-nucleotide polymorphisms was identified, flanked by primers *porbp2TMfwd* and *porb2TMrev* (Table 1). In five of these positions adenosine (A) or thymidine (T) residues in *P. o. curtisi* were replaced by cytosine (C) and guanidine (G) residues in *P. o. wallikeri* (Fig. 2A). We therefore postulated that PCR amplicons based on this region would differ in melt profile and designed a short amplicon to test this idea. Melt-curve analysis of amplicons detected in real-time by SYBR green incorporation, using a Corbett Rotorgene thermal cycler, confirmed that the peak melting temperature of the *P. o. curtisi* amplicon was consistently one degree lower than that of *P. o. wallikeri* in DNA from previously-characterised isolates of each species (Fig. 2Aa), and in isolates from two children in Uganda, collected within 2 days of each other in April 2009. *potra* PCR confirmed the species assignments made by the qPCR assay.

### 3.4 qPCR species discrimination in Uganda and Equatorial Guinea

For the 209 individuals surveyed in Luba and 110 from Punta Europa Scariba, Bioko Island, DNA was extracted from each sample and tested for the presence of *Plasmodium* spp. by LAMP (Polley et al., 2010), and all LAMP positives were further evaluated using species-specific nested PCR (Calderaro et al., 2007). In Luba, one person was infected with *P. falciparum* and *P.* *o. curtisi*, and two were infected with *P. falciparum* and *P.* *o. wallikeri*. One infected individual from Punta Europa Scariba harboured *P. falciparum*, *Plasmodium malariae* and *P. o. curtisi*.

In Apac District, *P. ovale* spp*.* infections were detected in six of 86 parasite positive blood samples of 241 clinic attendees. Ovale parasites were found either as mono-infections (*n *= 3), or as co-infections with *P. falciparum* (*n *= 2) or *P. falciparum* and *P. malariae* (*n *= 1). Two *P. ovale* spp. mono-species infections were incorrectly classified as *P. falciparum* infection by microscopy at densities of 320 and 360 parasites/μL. These six were comprised of five *P. o. curtisi* infections and one *P. o. wallikeri* infection.

*Plasmodium ovale* spp*.*-positive samples from the surveys in Buliisa and Mayuge Districts were tested using the qPCR discrimination assay. Of 1850 individuals tested for malaria parasites of any species, 30 were identified as harbouring *P. ovale*: 11 with *P. o. curtisi* and 19 with *P. o. wallikeri*. In each case, species identity was confirmed either by the *potra* amplicon polymorphism assay or (for two individuals) by sequencing the *pog3p P. ovale* glyceraldehyde-3-phosphatase (*pog3p*) locus as previously described (Sutherland et al., 2010).

Data from all sites are presented in Table 2.

## 4 Discussion

This study has extended previous reports of sympatry between the proposed species *P. o. curtisi* and *P. o. wallikeri* (Sutherland et al., 2010) to the Republic of Congo, Equatorial Guinea and specific locations in three regions of Uganda. The utility of gel-based and real-time qPCR-based amplification assays is demonstrated, deploying independent target sequences, for discrimination of the two parasite species from field samples collected on filter-paper. Furthermore, in five locations in Uganda and in one of two villages on Bioko Island, surveys taken at a single time provided examples of both species. Therefore we conclude there is no general physical or temporal barrier preventing recombination of these related parasite species, but rather that *P. o. curtisi* and *P. o. wallikeri* maintain their distinct genetic identity through as yet unknown biological mechanisms. These findings, which evaluated two independent loci for species discrimination, confirm the multigenic dimorphism of these two parasites across a broad geographical range, and thus further support the classification of *P. o. curtisi* and *P. o. wallikeri* as separate species.

The combined population prevalence of *P. ovale* spp*.* was found to be between 1% and 6%, in Uganda and Equatorial Guinea, and contributed alone or in combination with other *Plasmodium* spp. to between 6% and 8% of malaria infections. This is consistent with a recent analysis of 2588 malaria infections across Africa (Culleton et al., 2008), entomological and parasitological surveys in Guinea-Bissau (Snounou et al., 1993) and the finding of a higher than expected contribution of ovale malaria cases to imported disease in both Portugal and the UK (Snounou et al., 1998; Sutherland et al., 2010). We conclude that these two parasite species are both widespread and, at least in Africa, fairly common. The contribution of cryptic ovale infections, in the form of both latent liver hypnozoites and blood-stage infection below the level of PCR detection, is unknown but almost certainly indicates that the actual prevalence of *P. ovale* spp. infection in our study areas is higher than we have observed. The contribution of these parasites to human malaria infection has therefore been grossly over-looked, and it is hoped that the simple molecular methods for diagnosis and discrimination devised here will go some way to assist in providing more accurate estimates of the prevalence of ovale malaria in all endemic areas. Although it is now well established that ovale malaria occurs in Myanmar, Thailand, Vietnam and Cambodia (Tachibana et al., 2002; Win et al., 2004; Sutherland et al., 2010), the extent to which both *P. o. curtisi* and *P. o. wallikeri* have succeeded and spread through southeast Asia and the south western Pacific remains an open question. Further, as we have not analysed *potra* or *porbp2* sequences from parasites in this region, the discrimination methods used here now require validation on southeast Asian isolates. Of particular interest is the species diversity in Melanesian communities in Indonesia, Papua New Guinea and the Solomon Islands, where ovale malaria is likely to be widespread, if uncommon and under-reported (Sutherland et al., 2010).

The most likely reason for the stable genetic separation between *P. o. curtisi* and *P. o. wallikeri* remains an evolutionary one; that the two species represent separate transition events from non-human to hominid primates sometime in the last 1–3 million years (Sutherland et al., 2010). Such separate transitions would have given the two lineages a period of evolution in isolation from each other, and post-transition adaptation to the new host is unlikely to have proceeded in an identical fashion. It is expected therefore that the two species have accrued, through genetic drift, sufficient differences to prevent mating and recombination. The global distribution of *P. ovale* spp*.* throughout the tropics strongly suggests that any such polymorphisms will not be restricted by host ethnicity, as a wide range of human populations are known to be susceptible to ovale malaria.

One potential difference between *P. o. curtisi* and *P. o. wallikeri* may be in host erythrocyte preference and it is theoretically possible that host polymorphisms such as blood groups restrict the two parasites to separate human population compartments. This hypothesis is not supported by two earlier studies that found evidence of mixed infections of both types of *P. ovale* in three Thai malaria patients (Zhou et al., 1998; Tachibana et al., 2002). These studies detected genetic mixtures by sequencing of PCR amplicons from the small ribosomal subunit genes. However, neither analysis deployed a standard PCR method widely validated in other laboratories, and no quality control data or repeat results were presented that could rule out PCR contamination as the source of the mixed amplicons. Interestingly, Tachibana et al. (2002) went onto examine three additional dimorphic loci, *pos25*, *pos28-1* and *pos28-2*, in eight *P. ovale* patients but did not report any supporting evidence from these loci that a mixed type infection was present in the single individual identified as such by the ribosomal gene analysis. Thus the presence of mixed infections of *P. o. curtisi* and *P. o. wallikeri* in Thailand cannot be ruled out, but needs confirmation by multi-locus analyses such as those we have previously described (Sutherland et al., 2010).

An alternative explanation for the apparent lack of recombination between *P. o. curtisi* and *P. o. wallikeri* is that the two parasite species may differ in recognition molecules crucial for the mating process, such as the ookinete proteins studied by Tachibana et al. (2002). In this case it could be predicted that where both species are common, mixed infections will occasionally occur, but that these will not lead to successful cross-fertilisation and recombinant forms following subsequent mosquito transmission. Detailed epidemiological surveillance in Uganda, Nigeria and other areas where *P. o. curtisi* and *P. o. wallikeri* occur together in approximately equal proportions, may provide evidence that such mixed infections occur – this would support the findings in Thailand (mentioned previously in Section 4) and constitute strong evidence against the hypothesis that the two species are kept apart by mutually exclusive requirements for erythrocyte phenotype. Such continuing detailed surveillance will also provide the opportunity to further test our hypothesis that genetic recombination does not occur between the two forms of ovale malaria investigated here.

It is now important to focus on determining whether there are any important biological, epidemiological or clinical differences between the two *ovale* malaria parasites, such as relapse periodicity (Davis et al., 2001; Coldren et al., 2007), drug response or transmissibility to different mosquito species. Only a thorough understanding of these features can ensure that malaria elimination strategies, currently targeted at the greater threats posed by *falciparum* and *vivax* malaria (Greenwood et al., 2008), are also able to reduce or halt transmission of *P. o. curtisi* and *P. o. wallikeri*.

## Figures and Tables

**Table 1:** PCR Primer sequences and reaction conditions used for the amplification of sequences encoding *Plasmodium ovale* sp. tryptophan-rich antigen (PoTRA) and *P. ovale* reticulocyte-binding protein homologue (PoRBP2).

Gene	Primer name	Primer sequence	PCR product size (bp)	PCR cycling conditions (Thermocycler)
potra	PoTRA fwd3 PoTRA rev3	5′-GCACAAAAATGGTGCTAACC-3′ 5′-ATCCATTTACCTTCCATTGC-3′	787	95 °C for 2 min; (95 °C for 30 s; 58 °C for 45 s; 72 °C for 1 min) × 44 cycles; 72 °C for 5 min (TETRAD)
	PoTRA fwd5 PoTRA rev5	5′-ACGGCAAACCCGATAAACAC-3′ 5′-GTGTTTGTAGTATTTACAGG-3′	245 to 355	95 °C for 2 min; (95 °C for 30 s; 52 °C for 45 s; 68 °C for 1 min) × 44 cycles; 68 °C for 5 min (TETRAD)
porbp2	Porbp2TMfwd Porbp2TMrev	5′-TTGCAAACAAAAGTGCTCC-3′ 5′-CCTAATTCTCTTTGT(G/A)CCC-3′	120	95 °C for 15 min; (95 °C for 15 s; 53 °C for 30 s; 68 °C for 30 s) × 40 cycles; melt 55 to 95 °C, 0.5 °C steps, 5 s per step. (RG3000/RG6000)

**Table 2:** Prevalence^a^ of *Plasmodium ovale curtisi* (Poc) and *Plasmodium ovale wallikeri* (Pow) among sample sets investigated.

Site	Region, Country	Number and nature of samples investigated	Total with *Plasmodium*	Results (*potra* nested PCR)	Results (*porbp2* qPCR)	Ovale infections as proportion of all malaria	Population prevalence of ovale infections
Pointe-Noire 2007	Congo	2 clinical cases, ovale by microscopy		2 Pow			
Gamboma 2006	Congo	2 clinical cases, ovale by microscopy		2 Pow			
Brazzaville 2005–6	Congo	2 clinical cases, microscopy neg; ovale by PCR		2 Poc			
Luba	Bioko Island, Equatorial Guinea	209 survey	36 by LAMP	1 Poc 2 Pow	1 Poc 2 Pow	8.3%	1.4%
Punta Europa Scariba	Bioko Island, Equatorial Guinea	110 survey	13 by LAMP	1 Poc	1 Poc	7.7%	0.9%
Apac	Apac District, Uganda	241 all cause clinic attendees	89 by microscopy	5 Poc 1 Pow	5 Poc 1 Pow	6.7%	2.5%
Bugoigo	Buliisa District, Uganda	348 survey	202^b^	1 Poc; 3 Pow	1 Poc; 3 Pow	2.0%	1.1%
Walukuba	Buliisa District, Uganda	312 survey	155^b^	0 Poc; 1 Pow	0 Poc; 1 Pow	0.6%	0.3%
Piida	Buliisa District, Uganda	243 survey	141^b^	0 Poc; 0 Pow	0 Poc; 0 Pow	0.0%^c^	0.0% c
Bugoto	Mayuge District, Uganda	392 survey	264^b^	1 Poc; 4 Pow	1 Poc; 4 Pow	1.9%	1.3%
Bukoba	Mayuge District, Uganda	369 survey	279^b^	6 Poc^d^; 10 Pow^d^	6 Poc; 10 Pow	5.7%	4.3%
Lwanika	Mayuge District, Uganda	203 survey	124^b^	3 Poc; 1 Pow	3 Poc; 1 Pow	3.2%	2.0%
TOTAL		2427 (excluding Congo)	1303	18 Poc; 22 Pow	18 Poc; 22 Pow	3.1%	1.6%

*porbp2,* gene encoding *P. ovale* reticulocyte-binding protein 2, qPCR, quantitative PCR; LAMP, loop-activated amplification.

a As the precise sensitivity of our assays has not been determined, these are minimum estimates of prevalence.

b Denominator of individuals with parasitaemia determined by real-time PCR.

c No *P. ovale* sp*.* infections identified.

d For one individual with each species, no result was obtained in the *P. ovale* sp. tryptophan-rich antigen (*potra*) assay, but species designation was confirmed by *P. ovale* glyceraldehyde-3-phosphatase (*pog3p*) sequence analysis.
